# DNA Methylation in *LIME1* and *SPTBN2* Genes Is Associated with Attention Deficit in Children

**DOI:** 10.3390/children8020092

**Published:** 2021-01-29

**Authors:** Sung-Chou Li, Ho-Chang Kuo, Lien-Hung Huang, Wen-Jiun Chou, Sheng-Yu Lee, Wen-Ching Chan, Liang-Jen Wang

**Affiliations:** 1 Genomics and Proteomics Core Laboratory, Department of Medical Research, Kaohsiung Chang Gung Memorial Hospital and Chang Gung University College of Medicine, Kaohsiung 833, Taiwan; raymond.pinus@gmail.com; 2 Kawasaki Disease Center and Department of Pediatrics, Kaohsiung Chang Gung Memorial Hospital and Chang Gung University College of Medicine, Kaohsiung 833, Taiwan; 3 Department of Neurosurgery, Kaohsiung Chang Gung Memorial Hospital and Chang Gung University College of Medicine, Kaohsiung 833, Taiwan; ahonbob@gmail.com; 4 Department of Child and Adolescent Psychiatry, Kaohsiung Chang Gung Memorial Hospital and Chang Gung University College of Medicine, Kaohsiung 833, Taiwan; wjchouoe2@gmail.com; 5 Department of Psychiatry, Kaohsiung Veterans General Hospital, College of Medicine, Graduate Institute of Medicine, School of Medicine, Kaohsiung Medical University, Kaohsiung 813, Taiwan; shirleylee.ncku@gmail.com; 6 Department of Data Analysis, Natera Inc., San Carlos, CA 94070, USA; wenching.chan@gmail.com

**Keywords:** ADHD, epigenetic, neuropsychological test, DNA methylation, biomarker

## Abstract

DNA methylation levels are associated with neurodevelopment. Attention-deficit/hyperactivity disorder (ADHD), characterized by attention deficits, is a common neurodevelopmental disorder. We used methylation microarray and pyrosequencing to detect peripheral blood DNA methylation markers of ADHD. DNA methylation profiling data from the microarray assays identified potential differentially methylated CpG sites between 12 ADHD patients and 9 controls. Five candidate CpG sites (cg00446123, cg20513976, cg07922513, cg17096979, and cg02506324) in four genes (*LIME1*, *KCNAB2*, *CAPN9*, and *SPTBN2*) were further examined with pyrosequencing. The attention of patients were tested using the Conners’ Continuous Performance Test (CPT). In total, 126 ADHD patients with a mean age of 9.2 years (78.6% males) and 72 healthy control subjects with a mean age of 9.3 years (62.5% males) were recruited. When all participants were categorized by their CPT performance, the DNA methylation levels in *LIME1* (cg00446123 and cg20513976) were found to be significantly higher and those in *SPTBN2* (cg02506324) were significantly lower in children with worse CPT performance. Therefore, DNA methylation of two CpG sites in *LIME1* and one CpG site in *SPTBN2* is associated with attention deficits in children. DNA methylation biomarkers may assist in identifying attention deficits of children in clinical settings.

## 1. Introduction

Attention-deficit/hyperactivity disorder (ADHD) is a common neuropsychiatric disorder that is characterized by inattention, and/or excessive activity and impulsivity, [1]. This disorder affects approximately 5% of school-age children globally [2]. ADHD is generally considered to be a highly hereditary disease [3] and is a candidate disease for understanding the pathophysiology that underlies attention deficits. Epigenetic regulation refers to gene regulation that changes the phenotype without altering nucleotide sequence [4,5]. DNA methylation is a chemical modification of DNA that changes genetic expression without altering the DNA sequence and is a mechanism of gene regulation [6]. DNA methylation typically occurs in CpG dinucleotides, and the regions in which CpG aggregates are called CpG islands, such as the promoter region in the first exon. Previous studies have reported that prenatal stress could alter the brain function in offspring [7], and maternal diet could result in DNA methylation changes in offspring [8]. Moreover, DNA methylation changes may be associated with prenatal exposure to maternal anxiety or cigarette smoking [9,10].

DNA methylation that is related to inflammatory responses and the neuronal conduction of monoamines has been associated with ADHD [11,12]. A differential DNA methylation marker has been identified in *VIPR2* and *MYT1L* genes for ADHD [13,14]. The degree of methylation of the serotonin transporter gene (*SLC6A3* and *SLC6A4*) promoter is negatively correlated with the thickness of the right temporal lobe and the occipital cortex in ADHD patients and positively correlated with the impulsive symptoms of such patients [15,16,17]. Numerous studies have also reported that the degree of methylation of *DRD4* is significantly correlated with ADHD severity [18,19,20,21]. Rijlaarsdam et al. [22] pointed out that in cases of early-onset and persistent behavioral disorders, a prenatal unhealthy diet is related to more severe ADHD symptoms via higher IGF2 methylation. In addition, many genes and pathways were reported to be implicated in attention or intelligence-related disorders, including *NISCH*, *ST3GAL3* [23], *COMT*, *IFNG*, *MIR200B*, *SYNGAP1* [24,25], *BDNF* and *NGFR* [26]. A prospective meta-analysis which contained 2374 children DNA methylation (cg25520701) lies within the gene body of *CREB5* was associated with neurite outgrowth and an ADHD diagnosis [27].

Methylome-wide analysis is a hypothesis-free methodfor determining which genes are involved in the disease’s pathogenesis, which has the potential to find novel biological associations [28]. Several studies have used the Illumina Human Methylation 450K (Illumina 450k) array as a tool for methylome-wide analysis to detect ADHD methylomic biomarkers. Walton et al. [29] reported that DNA methylation across multiple genomic locations at birth differentiated ADHD trajectories. For example, *SKI* is involved in neural tube development; *ST3GAL3* is related to intellectual disability; *PEX2* is linked to peroxisomal processes, and *ZNF544* is implicated in ADHD. Heinrich et al. [26] analyzed methylation patterns in buccal cell DNA using the Illumina 450K array. CpG sites in the genes of the dopaminergic (*COMT*, *ANKK1*) and the neurotrophic (*BDNF*, *NGFR*) system are associated with Nogo-P3 and the severity of ADHD symptoms. Effects of methylation are related to both the functional aspects of cognitive control, motivation, and ADHD-related behaviors. Cecil et al. [30] examined genome-wide DNA methylation patterns using buccal swabs and identified themethylation levels of the *DRD4* gene as a potential biomarker of aggressive behavior.

The currently available genome-wide Illumina Infinium Human Methylation 850 BeadChip array (M850K) allows researchers to identify more than 850,000 methylation sites in a genome at the resolution of a single nucleotide and increases their ability to detect novel methylomic markers. No investigation has yet used the Illumina M850K to identify DNA methylation biomarkers of attention deficits. We performed the power calculation (α = 0.05, β = 0.8, allocation ratio N1/N2 = 1.5) and sample size would be 80 for patients and 54 for controls for detecting a moderate effect (0.5). We propose that DNA methylationsites identified using the M850K system can be a potential biomarker for detecting attention deficits among children. This work aims to identify potential DNA methylation biomarkers and to determine whether differentially expressed DNA methylation is associated with the characteristics of ADHD patients.

## 2. Materials and Methods

### 2.1. Study Participants

The research protocol (104-9250A3) was approved by the Institutional Review Board (IRB) at Chang Gung Memorial Hospital in Taiwan, and written informed consent was obtained from the parents or guardians of all participating children. The subjects of this study were eligible patients with ADHD treated in the outpatient Department of Child Psychiatry at Chang Gung Children’s Hospital in Taiwan along with healthy control children. The methods used in our previous research [31] were adopted. The criteria for selecting the ADHD patients included the following: (a) a clinical diagnosis of ADHD by a senior child psychiatrist based on the criteria provided in the Diagnostic and Statistical Manual of Mental Disorders, Fourth Edition, Text Revision (DSM-IV-TR), following structured interviews based on the Chinese version of the Schedule for Affective Disorders and Schizophrenia for School-Age Children, epidemiologic version (K-SADS-E) [31]; (b) kids aged between 6 and 16 years; (c) a Han Chinese ethnic background; (d) kids who have never taken any medications to treat ADHD. Patients suffering from major physical illnesses (such as genetic, metabolic, or infectious conditions) or having a history of comorbid major neuropsychiatric diseases (such as intellectual disabilities, autism spectrum disorder, bipolar disorders, major depressive disorders, psychotic disorders, substance use disorders, epilepsy, or severe head trauma) were excluded.

The control subjects consisted of healthy children undergoing health checkups and blood draws for laboratory examinations. The remaining blood samples of these subjects were applied for this study. The healthy control subjects were children who do not suffer from ADHD, are ethnically Han Chinese, between the ages of 6 and 16 years old, and from within the same catchment area. They have not suffered from any known major physical illness or any of the major neuropsychiatric diseases mentioned above.

### 2.2. Collecting Clinical Blood Samples and M850K Examination

Approximately 5 mL of blood was collected from each subject. Whole blood samples were placed in a centrifuge (3000 rpm for 10 min) and a red blood cell (RBC) lysis buffer (RBCBioscience) was added to remove the RBCs. Then, we collected DNA samples from the enriched total white blood cells (WBCs). The collected DNA samples were then subjected to bisulfite conversion with EZ DNA Methylation-Lightning ^TM^ Kit (Zymo Research, Irvine, USA). Next, the bisulfite converted DNA samples were subjected to Infinium Human Methylation 850 BeadChip array (M850K, Illumina, CA, USA).

The generated raw M850K data were first processed with GenomeStudio (Illumina, CA, USA) with the default parameters as suggested by a previous study [32]. Then, the GenomeStudio output data were subsequently analyzed with Partek Genomics Suite (Qiagen, CA, USA). Partek Genomics Suite first inputted the microarray data, followed by normalizing the data based on the Functional normalization method proposed by a previous study [33]. We had the samples divided into two sets, control, and disease. By comparing the two sets, we conducted one-way ANOVA and requested the criteria of a significant CpG dinucleotide as follows: FDR < 0.05 and beta value variation > 1.2. The FDR values were calculated based on the method proposed by Benjamini et al. (https://doi.org/10.1111/j.2517-6161.1995.tb02031.x). We also deal with the batch effect by specifying the “Remove Batch Effect” function of Partek. In addition, we also removed the CpG dinucleotides with missing beta values. We further also mapped the significant CpG dinucleotides back to the human genome (hg19) and determined whether they were located with the promoter regions (−5000 to +3000 base pair from transcription start site) based on the annotation of RefSeq 82.

### 2.3. Pyrosequencing and Sequence Analysis

DNA from WBC obtained from blood samples was used for pyrosequencing and sequence analysis. Pyrosequencing was conducted using the PSQ Gold SQA reagent kit (Qiagen) on a Pyromark Q96 ID platform (Qiagen, Hilden, Germany), according to the manufacturer’s instructions. Simply put, 50 μL of biotinylated PCR products were mixed with 3 μL of streptavidin sepharose beads (Amersham Biosciences AB, Umeå Västerbotten, Sweden) and 47 μL of binding buffer, and the mixture was then shaken at 2000 rpm for 10 min. A vacuum preparation tool was used to capture the immobilized complex. The single-strand purification was carried out to separate and discard the unbiotinylated strand. The beads that were bound to single biotinylated strands were released to a 96-well microtiter plate to which 49 μL of annealing buffer and 1 μL of complementary sequencing primer (pBR-V1.AS) had been added. The processed mixture was loaded onto the PyroMark ID system for pyrosequencing with 10 cycles of ATCG dispensing.

### 2.4. Neuropsychological Assessments

Conners’ Continuous Performance Test (Conners CPT) is a 14-min computerized test that primarily assesses attention and impulse control. [34] Briefly, participants are required to respond to stimuli on a computer screen by pressing a space bar whenever they see a letter that is not “X.”Several parameters are assessed. The Confidence Index (percentile) incorporates all CPT data that are obtained in the test in a probability stated as a number out of 100 that a significant attention problem exists. A lower T-score on the CPT represents better performance [35]. In this study, we categorized patients based on the Confidence Index of 50 into the better performance group (CPT ≤ 50) and the worse performance group (CPT > 50).

### 2.5. Clinical Measurements

A senior psychiatrist used the K-SADS-E diagnostic tool [31] to interview all participants in both the ADHD patient group and the control group. An experienced child psychologist used the Wechsler Intelligence Scale for Children–Fourth Edition (WISC-IV) [36] to evaluate individual patients in a room that was designed to reduce variability in testing conditions. The parents and a teacher of the patients were asked to complete the Swanson, Nolan, and Pelham Version IV Scale (SNAP-IV) parent form and SNAP-IV teacher form [37], respectively.

### 2.6. Statistical Analysis

Data were analyzed using the statistical software package SPSS, version 16.0 (SPSS Inc., Chicago, IL, USA). Variables were presented in terms of either mean (standard deviation) or frequency. Two-tailed *p*-values of < 0.05 were considered to indicate statistical significance. The Chi-square test or Fisher’s exact test was used to compare the gender distributions of the ADHD patients and the controls and those of the groups with CPT > 50 and CPT ≤ 50. Multivariate Analysis of Covariance (MANCOVA) was used to determine the potential difference in DNA methylation dinucleotides between the ADHD group and control, while the effect of age, sex, and intelligence quotient (IQ) were put under control.

## 3. Results

### 3.1. DNA Methylation Profile

**Figure 1:**
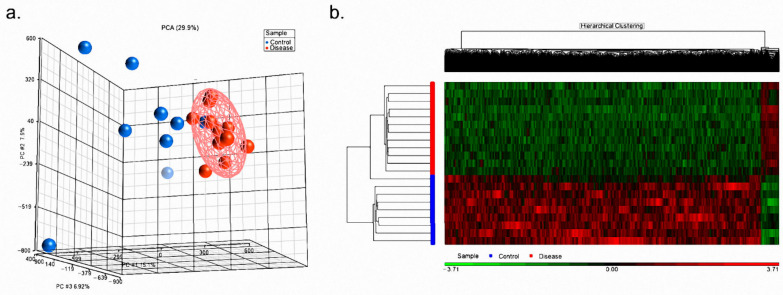
The overall DNA methylation patterns were derived from the M850K analysis. Blue and red dots (bars) denoted the control (healthy control) and disease (ADHD) subjects, respectively. (**a**) The PCA plot reflected the overall DNA methylation patterns of samples. The disease samples were aggregative more than the control samples. (**b**) The heat map reflected the variations of the significant CpG dinucleotides. Almost 95% of significant CpG dinucleotides were hypo-methylated in the disease set.

We had 9 control and 12 disease samples (age-and gender-matched) analyzed with M850K assays. After the GenomeStudio data was loaded, Partek initially generated the PCA plot. As shown in Figure 1a, the disease samples aggregated at a small space; while, the control samples scattered at a larger space. Such a result implied that the disease samples had more similar DNA methylation patterns than control samples did. Figure 1a also demonstrated that one control sample was closer to the disease samples than to the remaining control ones, which implied that it was more similar to the disease samples in terms of a DNA methylation pattern.

Under our criteria (FDR < 0.05 and beta variation > 1.2), 14,626 CpG dinucleotides were determined as significant based on ANOVA. Their variations (hyper-methylation or hypo-methylation) were demonstrated with the heat map (Figure 1b). Figure 1b demonstrated that the most significant CpG dinucleotides were hypo-methylated in disease samples and such result was pretty similar to previous studies. In inflammation-related diseases, such as Kawasaki disease [38] or autoimmune encephalitis [32], significant CpG dinucleotides tend to be hypo-methylated in disease samples. Figure 1b also illustrated that one control sample was clustered into the disease set which was consistent with the result of the PCA plot. The overall results of CpG methylation were shown with a Manhattan plot in Supplementary Figure S1.

By referring to the RefSeq 82 data, these significant 14,626 CpG dinucleotides were located at the putative promoter regions of 9749 genes. We also conducted pathway enrichment analysis on the 9749 genes (Supplementary Table S1). However, no obvious neuron-related pathway was enriched. Therefore, we investigated whether the genes hosted by significant CpG dinucleotides were associated with neurobiological processes, neurodevelopment, neurotransmission, or neuroprotection. As a result, a total of four genes (*LIME1*, *KCNAB2*, *CAPN9*, and *SPTBN2*) and five CpG sites (cg00446123, cg20513976, cg07922513, cg17096979, and cg02506324) were selected for further pyrosequencing analysis.

### 3.2. Pyro-Sequencing Analysis Result

Table 1 presents the characteristics of the ADHD patients and the healthy controls. A total of 126 ADHD patients (mean age 9.2 years, 78.6% males) and 72 healthy control subjects (mean age 9.3 years, 62.5% males) were recruited. ADHD patients were more likely than the control subjects to be male (*p* = 0.015) and to have a lower intelligence quotient (*p* < 0.001), higher inattention scores (*p* < 0.001), and higher hyperactivity/impulsivity scores (*p* < 0.001) as rated by parents or teachers; they also had worse values of the CPT performance, including Confidence Index (*p* < 0.001), Omission (*p* = 0.002), Commission (*p* = 0.039) and Detectability (*p* = 0.036) in the CPT. With respect to the four target genes (*LIME1*, *KCNAB2*, *CAPN9*, and *SPTBN2*) and the five CpG sites (cg00446123, cg20513976, cg07922513, cg17096979, and cg02506324), no significant difference in the levels of the CpG sites were identified between the ADHD patients and the controls (Table 2).

In our exploratory analyses, we further examined the levels of the CpG sites by comparing the CPT difference. Supplementary Table S2 summarized the characteristics of children with worse CPT performance (*n* = 115) and those with better CPT performance (*n* = 83). The group with the worse CPT group more likely to be male (*p* = 0.002), and to have higher inattention scores (*p* = 0.027) and higher hyperactivity/impulsivity scores (*p* = 0.018) as rated by parents, higher inattention scores (*p* = 0.003), and higher hyperactivity/impulsivity scores (*p* = 0.037) as rated by teachers, and worse values of the performance Confidence Index (*p* < 0.001), Omission (*p* < 0.001), Hit Reaction Time (*p* < 0.001) in the CPT.

After controlling for age, sex, or IQ (Supplementary Table S3), DNA methylation levels in *LIME1* (cg00446123 and cg20513976) were significantly higher, and those in *SPTBN2* (cg02506324) were significantly lower, in children with worse performance in the CPT. DNA methylation levels in *KCNAB2* (cg07922513) and *CAPN9* (cg17096979) did not differ significantly between children with worse and those with better CPT performance. We also tried a sex-stratified analysis and found that DNA methylation levels in *LIME1* (cg00446123) were significantly higher (*p*-value = 0.039) in boys with worse performance in the CPT. However, no significant difference in DNA methylation levels between different CPT performance and diagnosis group among girls.

## 4. Discussion

This work applied the M850K system to identify candidate DNA methylation markers for ADHD. A previous large epigenome-wide association study of childhood ADHD revealed peripheral DNA methylation (cg17478313 annotated to *SLC7A8* and cg21609804 annotated to *MARK2*) associated with disease and polygenic risk burden [39]. The study herein identified two novel DNA methylations, involving *LIME1* (cg00446123 and cg20513976) and *SPTBN2* (cg02506324), which are identified as effective in identifying attention deficits in children. 

The Lck interacting transmembrane adaptor 1 (*LIME1*) gene encodes a transmembrane adaptor protein that links the T and B-cell receptor to downstream signaling pathways through its association with the Src family kinases Lck and Lyn, respectively. Splicing of this gene yields multiple transcript variants [40]. The transcription start site of the *LIME1* gene, which has a key role in inflammatory pathways such as MAPK signaling, has been implicated in the pathophysiology of cerebral palsy [41]. The immune system may be affected by common genetic background and molecular mechanisms [42]. Prenatal exposure to inflammation is associated with changes in brain development in young offspring, including reductions in cortical gray matter volume and the volume of certain cortical areas [43]. The pathophysiology of ADHD is involved in morphological and functional brain abnormality, and modifications to neurotransmitter systems, including the dopaminergic, serotonergic, and glutamatergic systems [44]. Therefore, DNA methylation of the *LIME1* (cg00446123 and cg20513976) gene may influence that gene’s function and further affect the corresponding inflammatory pathway, which may be linked to the phenotype of attention deficits.

Spectrins are principal components of a cell’s membrane-cytoskeleton and comprise two alpha and two beta spectrin subunits. Spectrin beta non-erythrocytic 2 (*SPTBN2*) regulates the glutamate signaling pathway and is associated with neonatal cerebellar cortical degeneration [45]. Heterozygous missense variants in the *SPTBN2* gene, encoding the non-erythrocytic beta spectrin 2 subunit (beta-III spectrin), have been identified in autosomal dominant spinocerebellar ataxia type 5 (SCA5), which is a rare adult-onset neurodegenerative disorder that is characterized by progressive cerebellar ataxia [46,47,48,49]. The result herein reveals a potential association between *SPTBN2* and neurodevelopment. DNA methylation at the CpG sites of the *SPTBN2* gene may influence the process of neurodevelopment and be further linked to attention.

The interesting issue is that DNA methylation levels were not directly associated with ADHD diagnosis, but highly correlated with attention deficits as measured by the CPT. A diagnosis of ADHD depends on gathering information about behavioral symptoms from patients and their parents, teachers, or clinical observers. However, interviews and data collection are time-consumingand behavioral measures are vulnerable to rater and reporting biases [50]. The CPT is a computer-based neuropsychological test that is commonly used to measure an individual’s attention and impulsivity during a sustained task; it can be used in a clinical inquiry as part of a diagnostic procedure [51]. Numerous investigations have suggested that combining CPTs with an objective measure of activity may be particularly effective for clinical purposes [52,53]. We previously found that the association between levels of ADHD biomarkers and CPT performance was stronger than that with the severity of behavioral symptoms [54,55]. Consistent with the findings herein, the CPT may serve as an instrument for detecting the biological footprint and underlying pathophysiology of ADHD [56]. 

Studies have shown that the degrees of DNA methylation in peripheral blood and brain tissue are highly correlated [57]. Previous investigations have found DNA methylation markers in various genes, including the serotonin transporter gene (*SLC6A3*) [15], *SLC6A4* [16], *DRD4*, *5-HTT* [17,18,19,20,21,30], *VIPR2*, *MYT1L* [13], *5-HT3A* [17], *IFNG* [24], *COMT*, *ANKK1*, *BDNF*, *NGFR* [9], *NISCH* and *ST3GAL3* [23] genes. However, the aforementioned DNA methylation markers were not identified in this study. This discrepancy has numerous explanations. The first is that in previous studies, an illumine M450K system was used as an explorative tool as part of a target gene strategy, based on neurobiological theory. In contrast, this study is the first in which M850K is used to detect DNA methylation markers, and the fold change between patients and controls in our preliminary study was not large enough for detecting these markers. Second, some previous studies used saliva or other samples, whereas the DNA methylation information herein was obtained from WBC. DNA methylation may be tissue-specific, leading to variations among studies.

This investigation has several limitations. First, the HumanMethylation850K array captures a part of all CpGs in the genome. These polymorphisms reduce the quality of microarray-based measurements of DNA methylation and may limit the detection for DNA methylation–mediated effects of SNP on the phenotype. Second, the sample was small, and attention deficits co-occurred with the male gender. ADHD is generally heterogeneous and can be categorized into different subtypes, and psychiatric comorbidities can be observed in most ADHD patients. Owing to the smallness of the sample, whether gender or the heterogeneity of ADHD affects the results of DNA methylation was not addressed. Moreover, a MANCOVA analysis was used instead of a conditional logistic regression or logistic regression which may underestimate the standard errors [58]. Third, the DNA methylation information was obtained using total WBC. Although the degrees of DNA methylation in peripheral blood and brain tissue could be highly correlated [57], blood is unlikely to provide a complete picture of ADHD-related epigenetic processes, particularly in the brain. That is why the pathway or GO analyses based on the genes hosted by the significant CpG dinucleotides did not identify the neuron-related functions [27], including this study (Supplementary Table S3). Actually, the leukocyte population is composed of many different types of cells, including neutrophil, eosinophil, basophil, lymphocyte, and monocyte. In addition, lymphocyte can be further classified into b cell, CD4+ T helper cell, CD8+ cytotoxic T cell, regulatory T cell, natural killer cells, and so on. Recently, single-cell sequencing technology was invented to provide higher resolution into cell-type-specific patterns [59]. With the application of single-cell technology combined with bisulfite sequencing, we may enhance our understanding from total WBC level into leukocyte cell-specific level. By doing so, the methylation pattern from blood could better reflect disease. Fourth, the associations that were detected in this cross-sectional study may provide insights into the underlying biological process but not into causality. Future longitudinal studies of humans will help to overcome this issue. Finally, the participants in this study were all Han Chinese and recruited from a single hospital in Taiwan. Further research must be performed to determine whether our findings concerning DNA methylation markers can be generalized to various ethnicities or countries.

## 5. Conclusions

This work newly finds that DNA methylation at two CpG sites in *LIME1* (cg00446123 and cg20513976) and one CpG site in *SPTBN2* (cg02506324) is associated with attention deficits in children. DNA methylation biomarkers, while not associated with the diagnosis of ADHD, may help to identify attention deficits in clinical settings. Future studies are required to clarify the modulation of gene functions by DNA methylation and the biological mechanisms that underlie the attention modality.

## Figures and Tables

**Table 1:** Characteristics of Patients with ADHD and Healthy Controls.

Characteristics	ADHD (*N* = 126)	Controls (*N* = 72)	Statistic	*p*-Value
Sex			5.967	0.015 *
Male	99 (78.6)	45 (62.5)		
Female	27 (21.4)	27 (37.5)		
Age(years)	9.2 ± 2.1	9.3 ± 2.2	0.342	0.733
FSIQ of the WISC-IV	97.1 ± 10.5	108.3 ± 13.7	5.960	<0.001 *
Clinical measures				
SNAP-IV parent form (I)	16.4 ± 5.5	5.8 ± 6.0	12.921	<0.001 *
SNAP-IV parent form (H)	14.3 ± 6.5	4.5 ± 5.5	10.916	<0.001 *
SNAP-IV teacher form (I)	14.8 ± 5.9	4.5 ± 4.9	13.188	<0.001 *
SNAP-IV teacher form (H)	11.7 ± 6.9	2.9 ± 3.5	11.844	<0.001 *
**CPT**				
Confidence Index	62.5 ± 22.6	46.7 ± 21.0	4.966	<0.001 *
Omission	58.8 ± 18.1	51.6 ± 13.8	3.142	0.002 *
Commission	49.2 ± 9.8	46.2 ± 10.5	2.079	0.039 *
Hit Reaction Time	55.4 ± 12.9	55.5 ± 11.4	0.012	0.990
Detectability	51.2 ± 9.1	48.1 ± 10.9	2.109	0.036 *

Data are expressed as *N* (%) or Mean ± SD; FSIQ, Full-Scale Intelligence Quotient; WISC-IV, Wechsler Intelligence Scale for Children–Fourth Edition; I, inattention scores; H, hyperactivity/impulsivity scores; * *p* < 0.05.

**Table 2:** DNA Methylation Levels of Patients with ADHD and Healthy Controls.

Gene	CpG	ADHD (*N* = 126) Mean (SD)	Controls (*N* = 72) Mean (SD)	F	*p*-Value
*LIME1*	cg00446123+5	57.9 ± 9.4	58.9 ± 7.6	0.110	0.740
*LIME1*	cg00446123+9	50.6 ± 8.8	50.2 ± 7.8	0.054	0.817
*LIME1*	cg20513976	51.9 ± 8.0	51.6 ± 7.0	0.000	0.990
*LIME1*	cg20513976+5	58.6 ± 9.4	59.8 ± 8.2	0.832	0.363
*LIME1*	cg20513976+9	50.6 ± 9.0	50.0 ± 7.6	0.025	0.875
*KCNAB2*	cg07922513	49.5 ± 6.7	48.9 ± 5.7	0.295	0.588
*CAPN9*	cg17096979	78.6 ± 15.4	81.6 ± 15.7	0.963	0.328
*SPTBN2*	cg02506324	35.5 ± 5.5	35.2 ± 6.2	0.134	0.714

Owing to the design of the M850K microarray assay, each probe detects only one CpG dinucleotide. In addition, the neighboring probes keep a moderate distance to avoid mutual interference, which leaves many CpG dinucleotides locating between probes undetected. In this study, we used PyroMark to validate the methylation profiles of specific CpG dinucleotides by sequencing ~50 nt fragments. Therefore, the neighboring originally un-detected CpG dinucleotides could also be validated. For example, cg20513976+5 and cg20513976+9 were 5 and 9 base downstream apart from cg20513976, respectively.They were not detected with M850K assay but validated by PyroMark assay simultaneously with cg20513976. Data are expressed as F value and *p*-value, using MANCOVA, and controlling for age, sex, and Full Scale Intelligence Quotient of WISC-IV, Wechsler Intelligence Scale for Children–Fourth Edition.

## Data Availability

The data presented in this study are available on request from the corresponding author.

## Supplementary Materials

The following are available online at https://www.mdpi.com/2227-9067/8/2/92/s1, Figure S1: The Manhattan plot of all CpG dinucleotides, Table S1: Gene enrichment analysis on 9749 genes, Table S2: Characteristics of Children with CPT confidence index, Table S3: DNA Methylation Levels of Children with CPT confidence index.
