# Supplementary material for: DNA Methylation in LIME1 and SPTBN2 Genes Is Associated with Attention Deficit in Children
Source: Children (Basel). 2021 Jan 29;8(2):92. doi: 10.3390/children8020092 (PMC7912017; doi:10.3390/children8020092)
Supplement: Supplementary file 1 [file children-08-00092-s001.zip › Supplementary Files.docx]

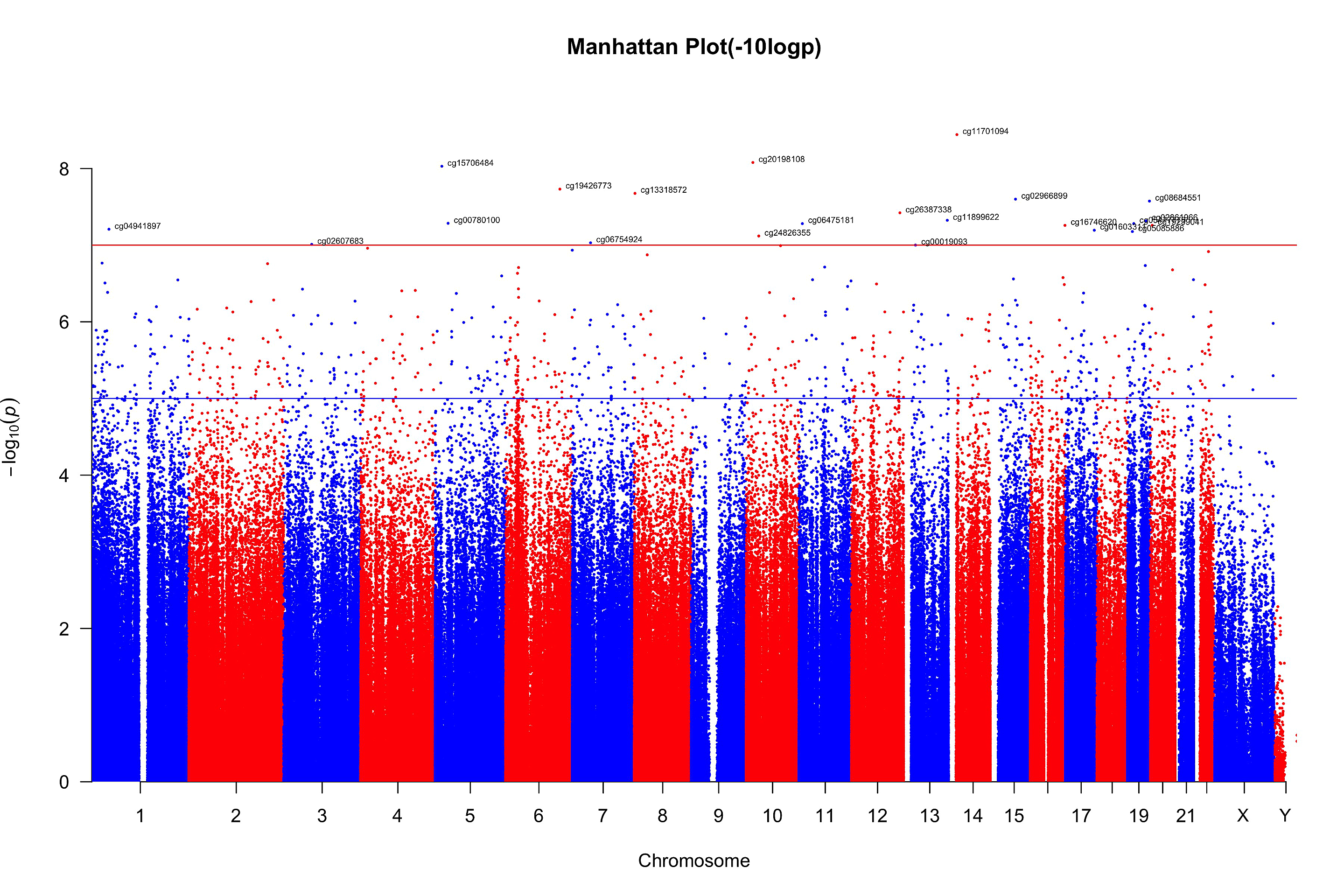


**Figure S1.** The Manhattan plot of all CpG dinucleotides. We used the Manhattan plot to present the p-values of all CpG dinucleotides analyzed. The CpG dinucleotides were arranged in the order of genomic positions within chromosomes. The Y-axis denoted the −log10 p-value.

**Supplementary Table S2**. Characteristics of Children with CPT confidence index >50 and ≤ 50

| **Characteristics** | **CPT > 50**  **(*N* = 115)** | **CPT** ≤ **50**  **(*N* = 83)** | **Statistic** | ***p*-value** |
| --- | --- | --- | --- | --- |
| **ADHD** |  |  | 10.491 | 0.001* |
| Yes | 84 (73.0) | 42 (50.6) |  |  |
| No | 31 (27.0) | 41 (49.4) |  |  |
| Sex |  |  | 9.170 | 0.002* |
| Male | 93 (80.9) | 51 (61.4) |  |  |
| Female | 22 (19.1) | 32 (38.6) |  |  |
| Age (years) | 9.1 ± 2.1 | 9.4 ± 2.2 | 1.022 | 0.308 |
| FSIQ of the WISC-IV | 99.8 ± 12.2 | 103.3 ± 13.7 | 1.872 | 0.063 |
| Clinical measures |  |  |  |  |
| SNAP-IV parent form (I) | 13.5 ± 7.5 | 11.0 ± 7.7 | 2.224 | 0.027* |
| SNAP-IV parent form (H) | 11.7 ± 7.5 | 9.1 ± 7.9 | 2.376 | 0.018* |
| SNAP-IV teacher form (I) | 12.3 ± 7.3 | 9.0 ± 7.3 | 3.039 | 0.003* |
| SNAP-IV teacher form (H) | 9.3 ± 6.9 | 7.1 ± 7.4 | 2.099 | 0.037* |
| **CPT** |  |  |  |  |
| Confidence Index | 71.1 ± 18.4 | 36.9 ± 11.8 | 15.931 | <0.001* |
| Omission | 63.4 ± 18.8 | 46.2 ± 5.2 | 9.341 | <0.001* |
| Commission | 47.4 ± 11.1 | 49.1 ± 8.6 | 1.154 | 0.250 |
| Hit Reaction Time | 59.8 ± 12.2 | 49.4 ± 9.6 | 6.497 | <0.001* |
| Detectability | 50.3 ± 10.8 | 49.7 ± 8.5 | 0.422 | 0.673 |

^a^ Data are expressed as N (%) or Mean ± SD; FSIQ, Full Scale Intelligence Quotient; WISC-IV, Wechsler Intelligence Scale for Children–Fourth Edition; I, inattention scores; H, hyperactivity/impulsivity scores; **p*<0.05

**Supplementary Table S3.** DNA Methylation Levels of Children with CPT confidence index >50 and ≤ 50.

| **Gene** | **CpG** | **CPT > 50 (N = 115)**  **Mean (SD)** | **CPT** ≤ **50 (N = 83)**  **Mean (SD)** | **F** | ***p*-value** |
| --- | --- | --- | --- | --- | --- |
| *LIME1* | cg00446123+5 | 59.5 ± 8.3 | 56.6 ± 9.1 | 5.077 | .025* |
| *LIME1* | cg00446123+9 | 52.0 ± 8.4 | 48.4 ± 8.1 | 8.535 | .004* |
| *LIME1* | cg20513976 | 52.9 ± 7.2 | 50.3 ± 8.0 | 5.872 | .016* |
| *LIME1* | cg20513976+5 | 60.3 ± 8.5 | 57.4 ± 9.3 | 5.301 | .022* |
| *LIME1* | cg20513976+9 | 51.7 ± 8.5 | 48.8 ± 8.3 | 5.089 | .025* |
| *KCNAB2* | cg07922513 | 48.4 ± 5.9 | 50.4 ± 6.7 | 3.592 | .060 |
| *CAPN9* | cg17096979 | 80.4 ± 15.6 | 78.9 ± 15.6 | .322 | .571 |
| *SPTBN2* | cg02506324 | 34.6 ± 5.6 | 36.4 ± 5.8 | 4.783 | .030* |

^a^ Data are expressed F value and p-value using MANCOVA, Controlling for age, sex and Full Scale Intelligence Quotient of WISC-IV, Wechsler Intelligence Scale for Children–Fourth Edition; **p*<0.05
